# Long non-coding RNA MRPS30 divergent transcript can be detected in the cytoplasm of triple-negative breast cancer cells and is targeted by microRNA-130b

**DOI:** 10.1080/21655979.2022.2031393

**Published:** 2022-02-22

**Authors:** Dongtao Wang, Qiang Song, Tianyong Zhao, Fang Wang, Yang Yu, Jing Qi, Pengfei Lyu, Xiangyang Duan

**Affiliations:** a Department of Oncological Surgery, Central Hospital, Baotou, Inner Mongolia, P.R. China; b Department of General Surgery, Central Hospital, Baotou, Inner Mongolia, P.R. China; c Department of Breast Surgery, The First Affiliated Hospital of Hainan Medical University, Haikou City, Hainan Province, PR. China; d Central Blood Station, Baotou, Inner Mongolia, P.R. China

**Keywords:** BRCAT54, miR-130b, TNBC, proliferation

## Abstract

Long non-coding RNA (lncRNA) MRPS30 divergent transcript (also known as BRCAT54) is recently reported to promote lung cancer. The involvement of BRCAT54 in triple-negative breast cancer (TNBC) is unknown. This study investigated the role of BRCAT54 in TNBC. The expression of BRCAT54 and microRNA(miR)-130b was detected by RT-qPCR. The subcellular location of BRCAT54 in TNBC cells was analyzed by nuclear fractionation assay. Overexpression of BRCAT54 and miR-130b was achieved in TNBC cells to explore the interaction between then. The role of BRCAT54 and miR-130b in TNBC cell proliferation was evaluated by BrdU assay. BRCAT54 was downregulated in TNBC, while miR-130b was upregulated in TNBC tissues. BRCAT54 and miR-130b were inversely correlated across both TNBC and normal tissues. BRCAT54 was detected in cytoplasm and was predicted to be targeted by miR-130b. In TNBC cells, downregulation of BRCAT54 was observed after the overexpression of miR-130b. Moreover, BRCAT54 decreased cell proliferation and miR-130b increased cell proliferation. Besides, BRCAT54 suppressed the role of miR-130b in increasing cell proliferation. Therefore, BRCAT54 can be detected in cytoplasm and was targeted by miR-130b to increase cell proliferation.

## Graphical abstract

**Figure:**
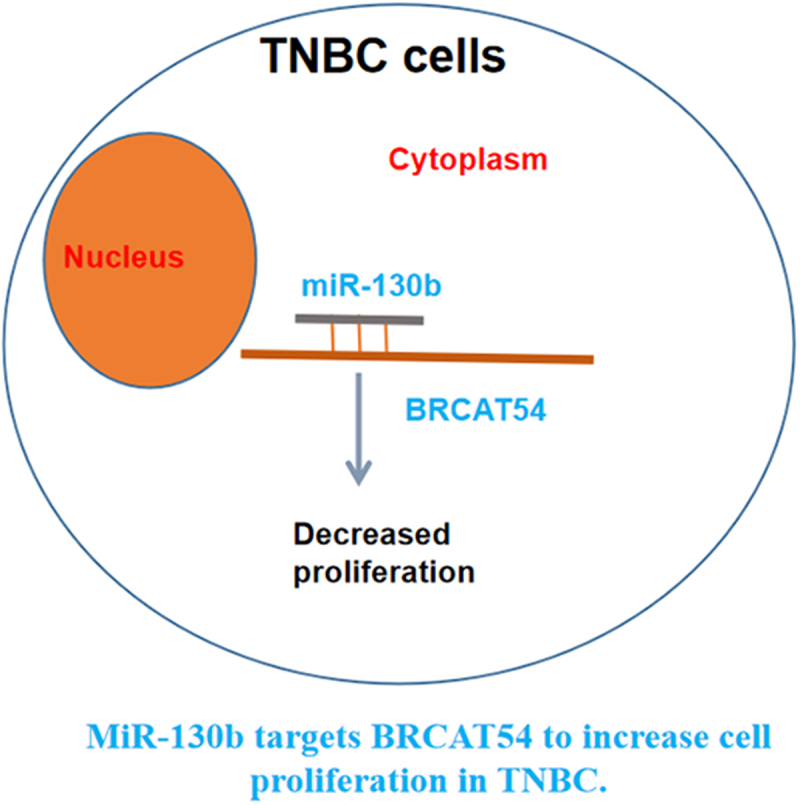

## Introduction

Breast cancer is the most common type of female malignancy and the second major cause of cancer deaths in females right after lung cancer [1]. The early symptoms include an abnormal mammogram or breast lump, which can be easily detected [2,3]. Therefore, most breast cancer cases are diagnosed with localized tumors and the prognosis is generally satisfactory [4]. However, breast cancer can be divided into different molecular subtypes that determine survival [5]. As a type of breast cancer that lacks the expression of common receptors, triple-negative breast cancer (TNBC) does not respond to commonly used receptor targeted therapy, such as HER2 targeted therapy [6,7], leading to poor prognosis. Therefore, the treatment of TNBC needs to be improved by more effective approaches. However, this attempt is limited due to the unclear mechanisms of this disease.

At present, TNBC is mainly treated by surgeries, such as lumpectomy and mastectomy, chemical drugs and radiation [6–9]. However, surgeries are not effective for patients with distant tumor metastasis, and chemical drugs and radiations cause severe side effects and recurrence is common [10,11]. Previous studies have shown that TNBC is related to a considerable number of molecular alterations, and some molecular factors with critical roles in the progression of TNBC can be targeted to treat TNBC [12]. Lacking the information of protein-coding, Long non-coding RNAs (lncRNAs) lack protein-coding capacity but participate in cancers by regulating protein synthesis [13]. Moreover, lncRNAs may sponge microRNAs (miRNAs) or targeted by miRNAs to regulate the progression of cancers and other human diseases [13]. Therefore, certain lncRNAs can be targeted to treat cancers including TNBC [14]. LncRNA MRPS30 divergent transcript (also known as BRCAT54) is recently reported to promote lung cancer [15]. The involvement of BRCAT54 in TNBC is unknown. Our preliminary analysis predicted that BRCAT54 could be targeted by a cancer-related miRNA, miR-130b [16]. Although the involvement of lncRNAs in TNBC has been reported by previous studies [17,18], the targeting of lncRNAs by miRNAs has not been well studied. We speculated that miR-130b might target BRCAT54 to participate in TNBC. This study was therefore carried out to explore the role of BRCAT54 in TNBC and its crosstalk with miR-130b.

## Materials and methods

### Clinical samples

TNBC and paired adjacent (within 3 cm of tumors) non-tumor tissues were collected from 60 TNBC patients who were admitted to the First Affiliated Hospital of Hainan Medical University between March 2018 and March 2020. The Ethics Committee of this hospital approved this study. The clinical data of the 60 patients were listed in Table 1. All patients signed the informed consent. All tissue samples were stored in a −80°C freezer prior to RNA isolation. All tissue samples were subjected to laser capture microdissection to isolate pure cells.

**Table 1.** Clinical data of 60 TNBC patients

Characteristics	n	Percentage (%)
Age		
≤50	30	50.0
>50	30	50.0
Lymphatic metastasis		
Positive	19	31.7
Negative	41	68.3
Tumor stage		
I–II stage	15	25.0
III–IV stage	45	75.0
Tumor size		
≤5 cm	27	45.0
>5 cm	33	55.0
Distant metastasis		
M0	41	68.3
M1	19	31.8

### Cell culture

Two human TNBC cell lines MDA-MB-231 and BT549 (Science Cell Laboratory) were used in this study. Fetal bovine serum was added into RPMI 1640 (100 μL/mL penicillin and streptomycin) to reach a final concentration of 10%. Cells were cultured at 37°C with 5% CO_2_.

### Transient transfections

Both MDA-MB-231 and BT549 cells were transfected with either pcDNA3.1- BRCAT54 vector or miR-130b mimic (Sigma-Aldrich). Transient transfections were conducted using the Neon Electroporation Transfection system (Thermo Fisher Scientific). The dosage of vector and miRNA was 8 μg vector and 500 nM mimic for 10^6^ cells, respectively. All cells were immediately washed for three times with fresh medium, followed by cell culture in fresh medium for further 48 h prior to the subsequent assays. Negative control (NC) cells were cells transfected by empty pcDNA3.1 vector or NC miRNA that targets no genes in the human genome. Control cells were cells without transfection.

### RNA extraction and process

After transfection, the cell medium was centrifuged at 3,000 g for 10 min. The supernatant was removed and cell pellets containing 10,000 cells were subjected to RNA isolation using RNAzol reagent (Sigma-Aldrich). Total RNAs were also isolated from 0.1 g tissue samples using the same method. Genomic DNA digestion was done with DNase I. A 2100 Bioanalyzer was used to analyze RNA integrity, and an RNA integrity number (RIN) higher than 8.1 (8.1–9.8) was achieved in all samples. Otherwise, RNA isolation was repeated until an RIN higher than 8 was obtained.

### RT-qPCR

Reverse transcription (RT) was performed with 1,000 ng total RNAs as the template, followed by qPCR (18S rRNA endogenous control) to determine the expression of BRCAT54. The expression of miR-130b was detected using the GeneCopoeia All-in-One™ miRNA qRT-PCR Reagent Kit (U6 as endogenous control). The 2^−∆∆CT^ method was applied to process data as an amplification rate close to 100% was reached for all primer pairs [19].

### Subcellular fractionation analysis

The Nuclear and Cytoplasmic Extraction Reagents (Thermo Fisher Scientific) were used to prepare cytoplasmic (C) and nuclear (N) extract from both MDA-MB-231 and BT549 cells. Cytoplasmic and nuclear fractions were separated by centrifugation at 3,000 g at 4°C for 10 min. Using the same methods mentioned above, total RNAs were isolated from both types of sample. Following RTs, Taq (NEB) was used to perform PCR to amplify BRCAT54. PCR products were ran on 1% agarose electrophoresis, followed by gel staining with EB. Images were taken under a MyECL imager (Bio-Rad).

### BrdU incorporation assay (proliferation assay)

Colorimetric immunoassay, which was based on the BrdU incorporation in DNA synthesis, was applied to analyze cell proliferation using the BrdU ELISA kit (Roche Diagnostics, Germany). Briefly, cells were collected at 48 h post-transfection, followed by incubation with medium containing 10 mM BrdU at 37°C for 2 h. After that, cell fixation was performed and peroxidase-conjugated anti-BrdU antibody was used to incubate the cells for 2 h. Next, 3,3′,5,5’-tetramethylbenzidine, which is a peroxidase substrate, was used to incubate the cells for 60 min. Finally, OD values at 370 nm and 492 nm were measured and BrdU incorporation was reflected by the value = absorbance at 370 minus absorbance at 492 nm.

### Statistical analysis

TNBC and paired non-tumor tissues were compared by paired t test. Unpaired t test was used for two independent group comparisons. ANOVA Tukey’s test was used to compare multiple groups. P < 0.05 was considered statistically significant.

## Results

### Analysis of the differential expression of BRCAT54 and miR-130b and their correlations

**Figure 1.**
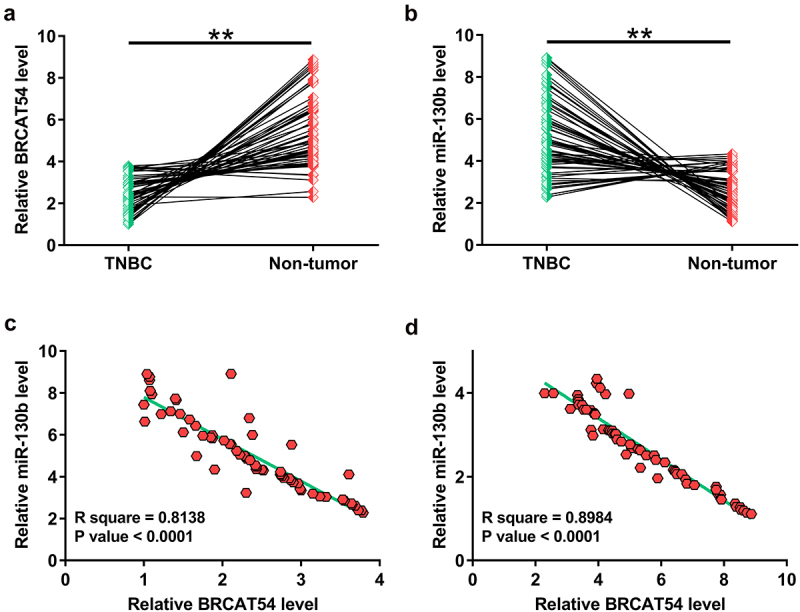
Analysis of the differential expression of BRCAT54 and miR-130b and their correlations.

Gene expression analysis is critical to predict functions. In this study, analysis of the expression of BRCAT54 and miR-130b in paired tissues from 64 TNBC patients was performed with RT-qPCR. The results showed that BRCAT54 was obviously downregulated in TNBC tissues (Figure 1a, *p*< 0.01), while miR-130b was highly upregulated in TNBC tissues compared to that in non-tumor tissues (Figure 1b, *p* < 0.01). Correlation analysis performed with Pearson’s correlation coefficient revealed a significant inverse correlation between the expression of BRCAT54 and miR-130b across TNBC tissues (Figure 1c). Similarly, they were also closely correlated across non-tumor tissues (Figure 1d). The close correlation between them suggested possible interactions between them.

The determination of BRCAT54 (A) and miR-130b (B) expression in paired tissues from 64 TNBC patients was analyzed by RT-qPCR. Correlation analysis with Pearson’s correlation coefficient was performed to analyze the correlation between BRCAT54 and miR-130b across TNCB tissues (C) and non-tumor tissues (D). **, *p* < 0.01.

### The subcellular location of BRCAT54 in TNBC cells and it may be targeted by miR-130b

**Figure 2.**
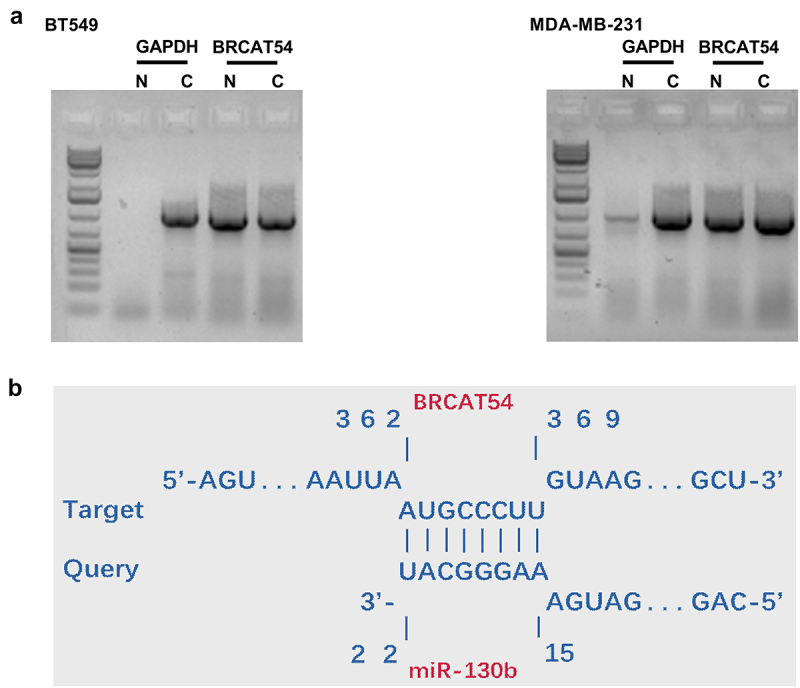
The subcellular location of BRCAT54 in TNBC cells and it may be targeted by miR-130b.

The function of RNAs is usually determined by their subcellular locations. Subcellular fractionation analysis was therefore performed to analyze the subcellular location of BRCAT54 in both BT549 and MDA-MB-231 cells. The results showed similar amount of RNA level of BRCAT54 in both cytoplasmic (C) and nuclear (N) samples. In contrast, in BT549 cells, the C marker GAPDH was only detected in C samples (Figure 2a). The targeting of BRCAT54 by multiple miRNAs was predicted by IntaRNA. It was observed that BRCAT54 was likely targeted by miR-130b (Figure 2b). Therefore, BRCAT54 may be targeted by miR-130b in cytoplasm.

Subcellular fractionation analysis was performed to analyze the subcellular location of BRCAT54 in both BT549 and MDA-MB-231 cells (A). The targeting of BRCAT54 by multiple miRNAs was predicted by miR-130b (B). C, cytoplasmic samples; N nuclear samples.

### The regulation of BRCAT54 by miR-130b in BT549 and MDA-MB-231 cells

**Figure 3.**
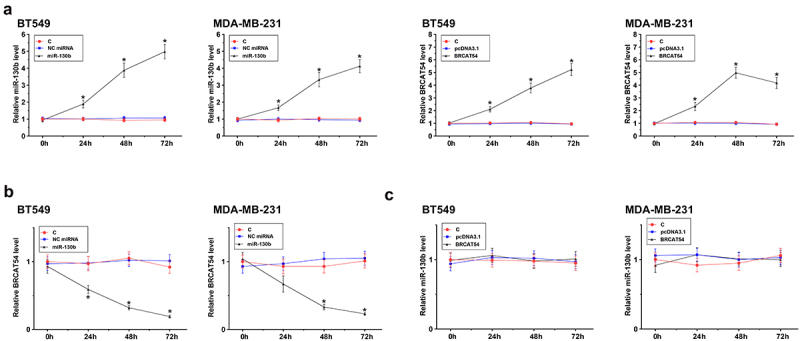
The regulation of BRCAT54 expression by miR-130b in BT549 and MDA-MB-231 cells.

To study the interaction between BRCAT54 and miR-130b, BT549 and MDA-MB-231 cells were overexpressed with BRCAT54 or miR-130b, and their overexpression was confirmed by RT-qPCR for three times with an interval of 24 h. Significantly enhanced expression was observed from 24 h to 72 h (Figure 3a, *p* < 0.05). In addition, downregulation of BRCAT54 was observed after the overexpression of miR-130b (Figure 3b, *p* < 0.05). Moreover, no significant alteration in the expression of miR-130b was observed after the overexpression of BRCAT54 (Figure 3c). Therefore, BRCAT54 was targeted by miR-130b in cytoplasm, leading to reduced RNA accumulation level.

BT549 and MDA-MB-231 cells were overexpressed with BRCAT54 or miR-130b, and their overexpression was confirmed by RT-qPCR three times with 24 h interval (A). The role of miR-130b in BRCAT54 expression (B) and the role of BRCAT54 (C) in miR-130b expression were analyzed by RT-qPCR to observe the alterations in gene expression. *, *p* < 0.05.

### Analysis of the role of BRCAT54 and miR-130b in the proliferation of BT549 and MDA-MB-231 cells

**Figure 4.**
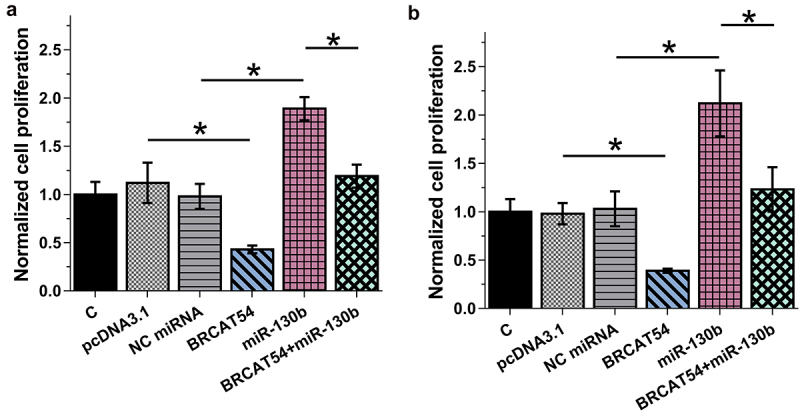
Analysis of the role of BRCAT54 and miR-130b in the proliferation of BT549 and MDA-MB-231 cells.

Cell proliferation contributed to cancer progression. BrdU incorporation assay was then performed to elucidate the role of BRCAT54 and miR-130b in the proliferation of BT549 and MDA-MB-231 cells. The results showed that BRCAT54 could decrease cell proliferation and miR-130b could increase cell proliferation. Besides, BRCAT54 suppressed the role of miR-130b in increasing cell proliferation (Figure 4, *p* < 0.05). Therefore, miR-130b might target BRCAT54 in cytoplasm to promote cell proliferation. It is worth noting that BRCAT54 and miR-130b did not affect cell apoptosis (flow cytometry, data not shown), stemness (flow cytometry, data not shown), invasion (Transwell, data not shown) and migration (Transwell, data not shown).

BrdU incorporation assay was carried out to elucidate the role of BRCAT54 and miR-130b in the proliferation of BT549 and MDA-MB-231 cells. *,p < 0.05.

## Discussion

The participation of BRCAT54 in TNBC and its potential crosstalk with miR-130b were first explored in this study. Our data suggested that BRCAT54 was downregulated in TNBC. In addition, BRCAT54 might be targeted by miR-130b in TNBC cells.

In a recent study, Yang *et al*. identified the expression and function of lncRNA BRCAT54 in non-small cell lung cancer [15]. Although BRCAT54 is upregulated in both plasma and tumor tissues of NSCLC patients, its upregulation plays a tumor suppressive role by regulating the JAK-STAT and calcium pathway genes to suppress tumor growth and metastasis [15]. This is an interesting observation because tumor suppressors are usually downregulated in cancer tissues. In this study, we showed the downregulation of BRCAT54 in TNBC and its inhibitory effects on TNBC cell proliferation. Our data and the previous study [15] showed that, although BRCAT54 may have different expression patterns in different cancers, it could function as a tumor suppressor.

MiR-130b shows different expression patterns and plays opposite roles in different cancers [16,20]. For instance, esophageal squamous cell carcinoma cells exhibit significantly increased expression levels of miR-130b, and the overexpression of miR-130b downregulates the expression of tumor suppressor PTEN, a major cell death inducer in cancer development, to promote tumor growth and progression [20]. In contrast, the downregulation of miR-130b in prostate cancer has been observed and overexpression of miR-130b can suppress tumor growth by downregulating MMP2 [21]. Based on our knowledge, the participation of miR-130b in TNBC is unknown. Our study showed the upregulation of miR-130b in TNBC and its enhancing effects on cell proliferation. Our data suggested the role of miR-130b as an oncogenic miRNA in TNBC. We also observed that BRCAT54 could be detected in cytoplasm. It has been well established that mature miRNAs are only detectable in cytoplasm. In addition, miR-130b was predicted to target BRCAT54, which was confirmed by overexpression assays. Therefore, miR-130b may target BRCAT54, a non-coding tumor suppressor RNA, to promote tumor growth in TNBC. However, further analyses, such as 5’-RACE sequencing and degradome sequencing, are needed to further confirm the targeting of BRCAT54 by miR-130b and identify the targeting site.

## Conclusion

In conclusion, BRCAT54 is downregulated in TNBC, and miR-130b may target BRCAT54 to promote TNBC cell proliferation.

## Data Availability

The data that support the findings of this study are available on request from the corresponding author. We accept reasonable request.
